# Monitoring the Coating of Single DNA Origami Nanostructures with a Molecular Fluorescence Lifetime Sensor

**DOI:** 10.1002/smll.202501044

**Published:** 2025-06-19

**Authors:** Michael Scheckenbach, Gereon Andreas Brüggenthies, Tim Schröder, Karina Betuker, Lea Wassermann, Philip Tinnefeld, Amelie Heuer‐Jungemann, Viktorija Glembockyte

**Affiliations:** ^1^ Department of Chemistry and Center for NanoScience Ludwig‐Maximilians‐Universität München Butenandtstr. 5‐13 81377 München Germany; ^2^ Max Planck Institute of Biochemistry and Center for NanoScience Am Klopferspitz 18 82152 Martinsried Germany; ^3^ Max Planck Institute for Medical Research Jahnstr. 29 69120 Heidelberg Germany

**Keywords:** coating, DNA origami, fluorescence lifetime imaging microscopy, nanoscale stability, polymer, sensing, silica, single‐molecule imaging

## Abstract

Protective coatings of functional DNA nanostructures with materials like silica or cationic polymers have evolved as a simple, yet powerful strategy to improve their stability even under extreme conditions. While over time, various materials and protocols have been developed, the characterization and quality assessment of the coating is either time consuming, highly invasive, or lacks detailed insights on single nanostructures. Here, a cyanine dye‐based molecular sensor is introduced to noninvasively probe the coating of DNA origami by either a cationic polymer or by silica, in real‐time and on a single nanostructure level. The cyanine dye reports changes in its local environment upon coating via increased fluorescence lifetime induced by steric restriction and water exclusion. Exploiting the addressability of DNA origami and the reversibility of the molecular sensor, the coating layer is probed at selected positions and in degrading conditions. Finally, the molecular sensor is combined with DNA PAINT super‐resolution imaging to investigate coating and structural integrity as well as preserved addressability of DNA nanostructures. The reported sensor presents a valuable tool to probe the coating of DNA nanodevices in complex biochemical environments in real‐time and at the single nanostructure level and aids the development of novel stabilization strategies.

## Graphical abstract

A molecular cyanine dye sensor reports on the coating of DNA origami with a polymer or silica by an increase in its fluorescence lifetime. The reversibility of the fluorescence lifetime shift enables the noninvasive real‐time probing of the coating even in degrading conditions. The molecular sensor can be combined with other single‐molecule imaging to simultaneously investigate the coating as well as structural and functional integrity of the nanostructure.

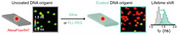

## 1 Introduction

DNA nanotechnology and, in particular, the DNA origami technique have advanced rapidly in the last decades and have reached an unprecedent level of complexity and functionality at the nanoscale.^[^ ^1^ ^]^ While the easy design of DNA origami and the spatial control of chemical modifications with base pair precision have opened up a plethora of potential applications in different fields, such as, plasmonics, biosensing, drug delivery, or nanorobotics, the intrinsic instability toward external factors often remains a bottleneck.^[^ ^2^ ^‐^ ^11^ ^]^

**Figure 1:**
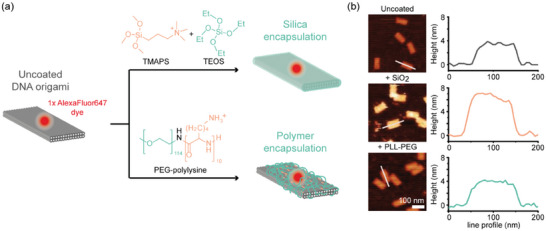
Principle of coating a DNA origami and its characterization using AFM. a) Scheme of two‐layer origami (TLO) with a single AlexaFluor647 label, coated by silica or by PLL‐PEG. b) Exemplary AFM scans and height‐profiles of uncoated and coated TLOs exhibit a measurable height increase only for coating by silica. Scale bar is 100 nm.

While DNA itself is susceptible to degradation by nucleases, employing it as building material in closely‐packed self‐assemblies necessitates the presence of specific cations like Mg^2+^ to compensate the anionic charge of the phosphate backbone, limiting the application window of DNA nanostructures to mild conditions (buffer conditions, specific ion concentrations, mild pH values, low temperatures, and irradiation) and to generally short device lifecycles.^[^ ^12^, ^13^, ^14^, ^15^, ^16^ ^]^ Consequently, multiple strategies have been developed to increase the stability of DNA self‐assemblies, e.g., by optimizing the design,^[^ ^17^ ^]^ by modifying the ends of staple strands with polymers such as polyethylene glycol (PEG),^[^ ^18^, ^19^ ^]^ by covalently connecting neighboring thymine bases via UV light‐induced cross‐linking,^[^ ^20^, ^21^, ^22^ ^]^ or by replacing defective staple strands via dynamic self‐repair.^[^ ^23^ ^]^ A simple, yet extremely effective strategy to increase the stability of functional DNA nanodevices is protective coating via electrostatic interactions between the negatively charged phosphate backbone of the DNA and a positively charged coating agent (**Figure** **1**a). Using the cationic silica precursor *N*‐[3‐(trimethoxysilyl)propyl]‐*N,N,N*‐trimethylammonium chloride (TMAPS) in a mixture with the classic precursor tetraethyl orthosilicate (TEOS) enables the growth of nanometers thick silica layers on DNA origami, either in solution or immobilized on various substrate surfaces.^[^ ^24^, ^25^, ^26^ ^]^ In a similar approach, DNA nanostructures can be coated with cationic polymers, such as poly‐l‐lysine polyethylene glycol block copolymer (PLL‐PEG), leading to a sub‐nanometer thick shell.^[^ ^27^, ^28^, ^29^ ^]^ While the silicification of DNA nanostructures leads to highly increased mechanical, chemical, biological and thermal stability,^[^ ^25^, ^30^, ^31^ ^]^ the coating with PLL‐PEG increases the chemical stability in low‐salt and serum conditions and it can be reversed by the addition of an anionic polymer such as dextran sulfate.^[^ ^27^, ^28^ ^]^ Despite coating with a thick silica shell or a PLL‐PEG layer, DNA docking sites remain accessible and addressable enabling DNA binding assays even in degrading conditions.^[^ ^32^, ^33^ ^]^ While the highly improved stability and preserved functionality of coated DNA nanostructures broadens the scope of applications, the verification of the coating process and its quality is still time‐consuming, highly invasive or rather indirect. So far, the silicification of DNA origami has been investigated either by transmission electron microscopy (TEM), atomic force microscopy (AFM), or X‐ray spectroscopy techniques such as energy dispersive X‐ray spectroscopy or small‐angle X‐ray scattering.^[^ ^24^, ^25^, ^26^, ^30^, ^31^, ^33^ ^]^ While the sub‐nanometer thick PLL‐PEG coating is hardly visible in AFM (Figure 1b), it can be characterized by TEM or probed by gel electrophoresis, as DNA origami completely covered with cationic polymer lose their charge and electrophoretic mobility.^[^ ^27^, ^28^, ^29^, ^32^ ^]^ While gel electrophoresis is the most commonly used quality check for the coating with PLL‐PEG, this technique remains blind to aggregates, that can occur during coating in solution, also resulting in a suppressed electrophoretic mobility.^[^ ^27^, ^29^ ^]^ Imaging techniques such as TEM, AFM or X‐ray spectroscopy, on the other hand, are highly invasive or time‐consuming preventing a quick and easy characterization of coated nanostructures under application conditions. Leveraging the noninvasive nature and sensitivity of single molecule fluorescence imaging, here, we report a novel strategy to study the coating of DNA nanodevices on a single structure level.

Since a coating layer increases the local viscosity and at the same time decreases the steric freedom of chemical modifications on the DNA origami, we reasoned that labeling a DNA nanostructure with an environment‐sensitive fluorophore at selected positions could enable probing of the coating process with nanometer precision on a single nanostructure level. Cyanine dyes are highly sensitive to their microenvironment since steric hindrance or a high viscosity slows down the photoisomerization from the emissive *trans* to the nonemissive *cis* state resulting in an increased fluorescence lifetime and photon count rate.^[^ ^34^, ^35^ ^]^ This effect has been exploited to study the binding of proteins or nucleic acids in the closed vicinity of a cyanine dye and has been termed protein‐induced (or photoisomerization‐related) fluorescence enhancement (PIFE).^[^ ^36^, ^37^, ^38^ ^]^ Additionally, a fluorophore embedded in the coating layer is also potentially less accessible for solvent molecules. For silicification, the displacement of more than 40% of the internal hydration water has been reported, indicating a strong hydrophobic condensation effect within silicified DNA nanostructures.^[^ ^31^ ^]^ Water has been shown to quench the fluorescence of red‐emitting dyes (absorption and emission > 600 nm) via a resonant energy transfer from the excited S_1_ state of the fluorophore to harmonics and combination bands of OH vibrational modes in the H_2_O molecule.^[^ ^39^ ^]^ Quenching can be suppressed by replacing water with its heavy analogue D_2_O leading to an increased fluorescence lifetime and photon count rate of the dye.^[^ ^40^, ^41^ ^]^ This effect has been exploited to sense the number of water molecules in the hydration sphere of red‐emitting dyes encapsulated in reverse micelles.^[^ ^42^ ^]^ Expecting that a coating layer on a DNA origami changes the local environment and decreases the concentration of water molecules in the hydration sphere of an embedded fluorophore, we reasoned that the red‐emitting cyanine dye AlexaFluor647 (AF647) could be a suitable candidate as a molecular sensor for the coating process. To remain independent of laser and setup fluctuations, we chose the fluorescence lifetime as a noninvasive readout to investigate the in situ coating of single, AF647 labeled DNA origami nanostructures with PLL‐PEG and silica. While the hereby expected fluorescence lifetime shift can be used as a measure of a restricted environment of the fluorophore and decreased water quenching, the width of the lifetime distribution might be indicative of the heterogeneity of the coating around the dye. We demonstrate the feasibility of the molecular sensor to probe the in situ coating at different positions on the immobilized nanostructure and its stability in degrading conditions (low ionic strength, degrading enzymes), even without photostabilization or without time‐consuming post processing. Finally, we combine the fluorescence lifetime sensor with DNA points accumulation for imaging in nanoscale topography (DNA‐PAINT) super‐resolution imaging to simultaneously probe the coating layer, the structural integrity of coated DNA origami and the retained addressability of DNA docking sites.

## 2 Results

To test, whether the designed molecular fluorescence lifetime sensor can probe the coating on DNA nanostructures, we designed a two‐layer DNA origami nanostructure (TLO, Figure 1a and Figure S1, Supporting Information). The TLOs were equipped with multiple biotin‐labeled staple strands to enable immobilization on neutravidin or streptavidin functionalized glass slides and were labeled with environment‐sensitive AF647. Before investigating the fluorescence lifetime of the cyanine dye, we first aimed to verify the successful coating of immobilized TLO nanostructures by PLL‐PEG or silica via AFM as previously reported for silicified DNA origami (see Figure S2a, Supporting Information).^[^ ^25^, ^26^, ^30^, ^33^ ^]^ Uncoated TLO exhibited a height of around 4 nm, while silicification led to a height increase of around 2 nm. In the case of the PLL‐PEG coating, no significant height increase was visible in the AFM scans, either because the coating shell is too thin (sub‐nanometer thickness as measured in cryo‐EM studies) or not rigid enough to be measured by the AFM cantilever. Nevertheless, the successful coating by PLL‐PEG could be confirmed by subsequent incubation in degrading low salt conditions (Mg^2+^ free and EDTA‐containing buffer). While uncoated TLO degraded and collapsed into rod‐shaped debris, the PLL‐PEG and silica coated nanostructures both remained intact, indicating successful protection for PLL‐PEG coating otherwise undetectable in AFM imaging (see Figure S2b, Supporting Information).

**Figure 2:**
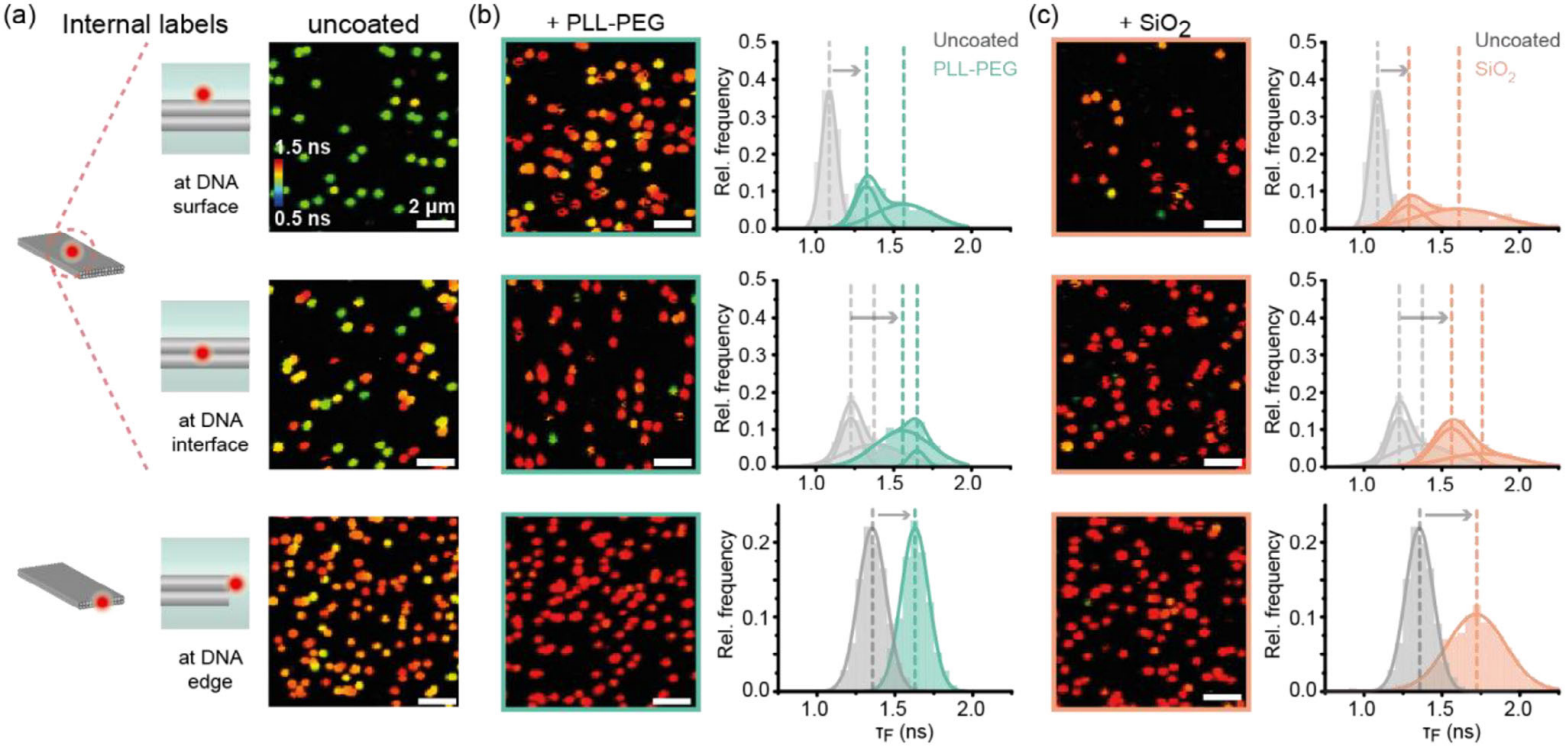
Characterization of the fluorescence lifetime‐based sensor to probe the in situ coating at different positions on immobilized DNA origami nanostructures by directly modifying selected staple strands (internal label). a) Different internal labeling positions of the AF647 fluorophore on the TLO (left). FLIM scans of Alexa647‐labeled TLO origami after immobilization (right). b) FLIM scans after coating with PLL‐PEG and spot‐integrated lifetime distributions. c) FLIM scans after silicification and spot‐integrated lifetime distributions. Dotted lines indicate mean values of Gaussian fits. Sample sizes: top row—752 molecules for uncoated, 1420 molecules for PLL‐PEG coated and 529 molecules for silica coated TLOs. Middle row—1082 molecules for uncoated, 960 molecules for PLL‐PEG coated and 1659 molecules for silica coated TLOs. Bottom row—1931 molecules for uncoated, 2357 molecules for PLL‐PEG coated and 1996 molecules for silica coated TLOs.

Next, we investigated the fluorescence lifetime of the cyanine label at different positions on uncoated and coated DNA nanostructures. To this end, we immobilized the nanostructures on biotinylated BSA and NeutrAvidin functionalized microscope glass slides and coated them with PLL‐PEG or silica. Single‐molecule fluorescence lifetime imaging microscopy (FLIM) scans were acquired on a custom‐built confocal microscope with a time‐correlated single photon counting unit (TCSPC).^[^ ^43^ ^]^ Acquired FLIM scans were analyzed by picking individual nanostructures and extracting the spot‐integrated fluorescence lifetime information. Distributions of the obtained fluorescence lifetime values were then fitted by Gaussian distribution functions, to determine the mean of each individual distribution. First, we labeled the TLO nanostructure internally with a single AF647, i.e., directly at the 3′‐end of a selected staple strand, to ensure that the sensor dye is embedded in the coating layer. To probe the coating process at the surface, inside and at the edge of the DNA nanostructure, we placed the sensor dye internally at the upper DNA surface, internally at the interface of the two DNA layers or at the end of a DNA origami helix (**Figure** **2**a). FLIM scans of uncoated DNA origami (Figure 2a) revealed that the three labeling positions result in different microenvironments of the AF647 label and consequently in different initial fluorescence lifetime distributions. The fluorescence lifetime distribution of the internal sensor label at the upper surface of the TLO origami revealed a sharp peak around 1.08 ± 0.05 ns close to the reported fluorescence lifetime of a free AF647 dye (Figure 2a, first row). The fluorescence lifetime distribution of the internal label at the DNA interface within the TLO design, on the other hand, revealed two populations with higher fluorescence lifetimes (for fitted values see Table S5, Supporting Information) indicating a more complex environment of the dye within the DNA origami with more steric restriction and possible interactions with the DNA origami backbone. Placing the FLIM sensor at the end of a helix on the edge of the TLO resulted in a single population of higher initial fluorescence lifetimes indicating a homogenous yet more restricted environment when compared to the DNA origami interface. As proposed in our sensor design, coating with PLL‐PEG (Figure 2b) or silica (Figure 2c) resulted in similar, significant increase in the fluorescence lifetime of AF647 for all three labeling positions of at least 0.2 ns (Table S5, Supporting Information). For the AF647 label at the surface of the TLO, the initially single Gaussian distribution was shifted by both coating agents to two populations of higher fluorescence lifetimes indicating two different microenvironments of the dye after the formation of the protective layers. For the TLO labeled with AF647 internally at the DNA interface, both coating agents induced a shift of the initial two fluorescence lifetime populations to higher fluorescence lifetimes. Consistent with the increase in fluorescence lifetime, the intensity of AF647 also increased after both coating processes (Figure S3, Supporting Information). The comparable significant fluorescence lifetime shifts for both coating agents indicate that our molecular sensor design works independently of the coating material and can be employed to probe coating‐induced effects, such as steric restriction or water repulsion, at different positions on or inside the DNA nanostructure with a high spatial control.

To exploit the in situ sensing ability and to better understand the coating process we studied the different DNA origami coating strategies over time (Figures S4 and S5, Supporting Information). Already after 30 min, we observed a complete fluorescence lifetime shift when the TLO nanostructures were incubated with PLL‐PEG indicating the rapid formation of the protective polymer layer. A complete fluorescence lifetime shift for silicification, on the other hand, required an incubation of the precursor solution for at least 24 h, which is in agreement with previously reported silicification kinetics.^[^ ^31^ ^]^ To obtain absolute lifetime values, the acquired lifetime decay curve of every picked fluorescent spot in the FLIM scans representing a single DNA origami nanostructure was re‐convoluted with the measured instrument response function (IRF) of the confocal microscope (exemplary TCSPC decay curves in Figure S6, Supporting Information). As the absolute lifetime shift induced by the coating layers was unaffected by the re‐convolution step (Figure S7, Supporting Information), spot‐integrated lifetime populations were used throughout this study to highlight the fast and straight‐forward readout of the coating process (for more details see Section 1.10, Supporting Information). While FLIM imaging was performed in a photostabilization buffer (see Materials and Methods section for details) to obtain high photon numbers, the fluorescence lifetime shift after coating with PLL‐PEG or silica could also be obtained from FLIM scans performed without photostabilization (Figure S8, Supporting Information). This allowed for probing the coating process of DNA origami in situ and in different application conditions without the need of specialized imaging buffer.

**Figure 3:**
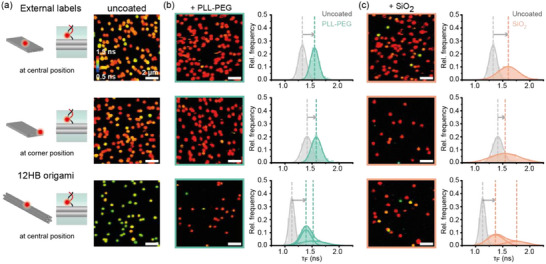
Characterization of fluorescence lifetime‐based sensor to probe the in situ coating at different positions on immobilized DNA origami nanostructures by externally binding a fluorescently modified imager strand via hybridization (external label). a) Different labeling positions of a single AF647 fluorophore on the TLO (left). FLIM scans of Alexa647‐labeled TLO origami after immobilization (right). b) FLIM scans after coating with PLL‐PEG and spot‐integrated lifetime distributions. c) FLIM scans after silicification and spot‐integrated lifetime distributions. Dotted lines indicate mean values of Gaussian distribution fits. Sample sizes: top row—2202 for uncoated, 2004 for PLL‐PEG and 1883 molecules for silica coated TLOs. Middle row—1744 for uncoated, 1575 for PLL‐PEG and 810 molecules for silica coated TLOs. Bottom row—1499 for uncoated, 663 for PLL‐PEG and 1261 for silica coated TLOs.

We further aimed to probe the coating process at different labeling positions using a more modular and less costly labeling strategy. To this end, we used a 3′‐AF647‐labeled, 21‐nt oligonucleotide which can hybridize externally to a complementary ss‐DNA extension positioned on DNA origami. In this manner, a single fluorescently labeled oligonucleotide is sufficient to screen the presence of the coating at different positions on various nanostructures making the approach more cost‐effective (**Figure** **3**). We then probed the homogeneity of the coating layers at different positions on the TLO by positioning the DNA docking sites for external labelling either in the center or in a corner of the upper DNA origami surface. To underline that our molecular sensor can be applied to any DNA origami nanostructure, we also studied AF647 labeled 12 helix bundle (12HB) DNA origami, which is based on honeycomb lattice in contrast to the square lattice of TLO.

Using the external labeling approach at different positions on the DNA origami led to different microenvironments of the cyanine dye and, in turn, different fluorescence lifetime distributions for uncoated DNA origami (Figure 3a). While AF647 labels bound externally to uncoated TLO exhibited higher lifetimes (Table S5, Supporting Information) than the internal dye at the DNA surface, indicating an already higher restricted microenvironment on the external DNA docking sites, the external cyanine label on the 12HB origami exhibited a lower fluorescence lifetime distribution indicating a relatively free microenvironment. Again, coating with both PLL‐PEG and silica induced similar shifts in the fluorescence lifetime distributions independently of the labeling position or DNA origami design (Table S5, Supporting Information). While revealing different fluorescence lifetimes for uncoated TLO origami, both external cyanine labels showed similar fluorescence lifetime populations after coating with both silica or PLL‐PEG. The external label on the 12HB DNA origami, on the other hand, revealed two fluorescence lifetime subpopulations after coating with either PLL‐PEG or silica indicating a more restricted and heterogenous microenvironment of the dye than before the coating. Except for the case of coated 12HB nanostructures, the external labels generally revealed unimodal distributions indicating simpler binding situations than for the internal labels, as shown in Figure 3.

**Figure 4:**
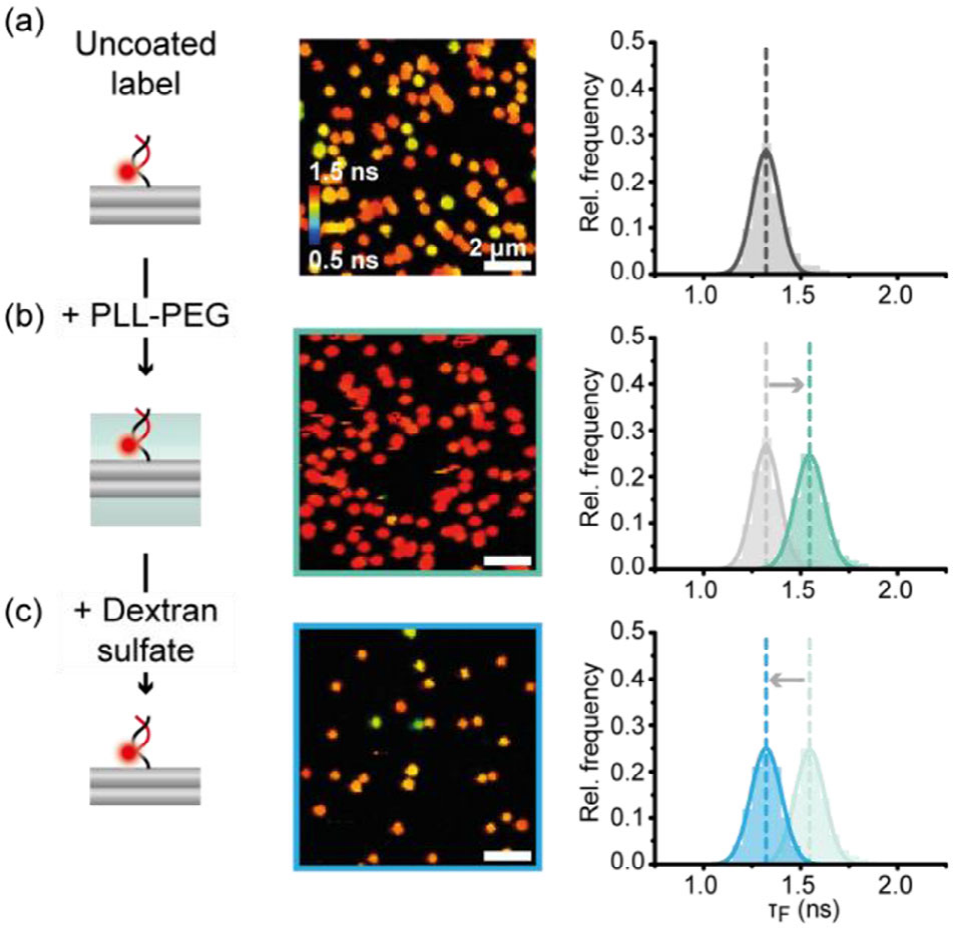
Probing the reversible coating with PLL‐PEG and dextran sulfate on a TLO nanostructure with an external label. a) FLIM scan and fluorescence lifetime distribution for uncoated TLO with the externally labeled sensor dye. b) FLIM scan and spot‐integrated fluorescence lifetime distribution for PLL‐PEG coated TLO with the externally labeled sensor dye reveals a lifetime shift to higher values. c) FLIM scan and spot‐integrated fluorescence lifetime distribution for initially PLL‐PEG coated TLO from b) reveals a quantitative shift back to a lifetime distribution corresponding to uncoated TLO nanostructures. Sample sizes of 2202 molecules for uncoated, 2004 molecules for PLL‐PEG coated and 870 molecules for de coated TLOs.

After successfully employing internal and external dye labeling strategies to probe the coating of DNA nanostructures at different positions, we went on to test the reversibility of the FLIM sensor by probing the fluorescence lifetime after the coating with PLL‐PEG and after subsequent removal of the polymer coating by the addition of an anionic polymer. To this end, PLL‐PEG coated TLO nanostructures were incubated with anionic dextran sulfate, which has been reported to decomplex and thus remove the cationic polymer coating.^[^ ^28^ ^]^ Indeed, the fluorescence lifetime distribution of PLL‐PEG coated TLO with an external cyanine label at the center of the upper surface revealed a quantitative shift back to the initial lifetime distribution of the uncoated nanostructure (**Figure** **4**). Accordingly, we also observed a reversible shift of the fluorescence lifetime for the molecular sensors at internal label positions on the upper surface and at the interface inside the TLO design (Figure S9, Supporting Information) highlighting the reversibility of the molecular sensor and the polymer coating independently of the labeling position. A complimentary investigation via AFM revealed structural integrity of the TLO origami after removal of the PLL‐PEG coating even though losing a majority of immobilized nanostructures upon addition of the anionic dextran sulfate (Figure S10, Supporting Information). Furthermore, we studied the decomplexation process in real‐time by scanning the same field of view over time after addition of dextran sulfate and saw a complete shift back to uncoated nanostructures within the first 5 min highlighting the fast kinetics of this process (Figure S11, Supporting Information).

**Figure 5:**
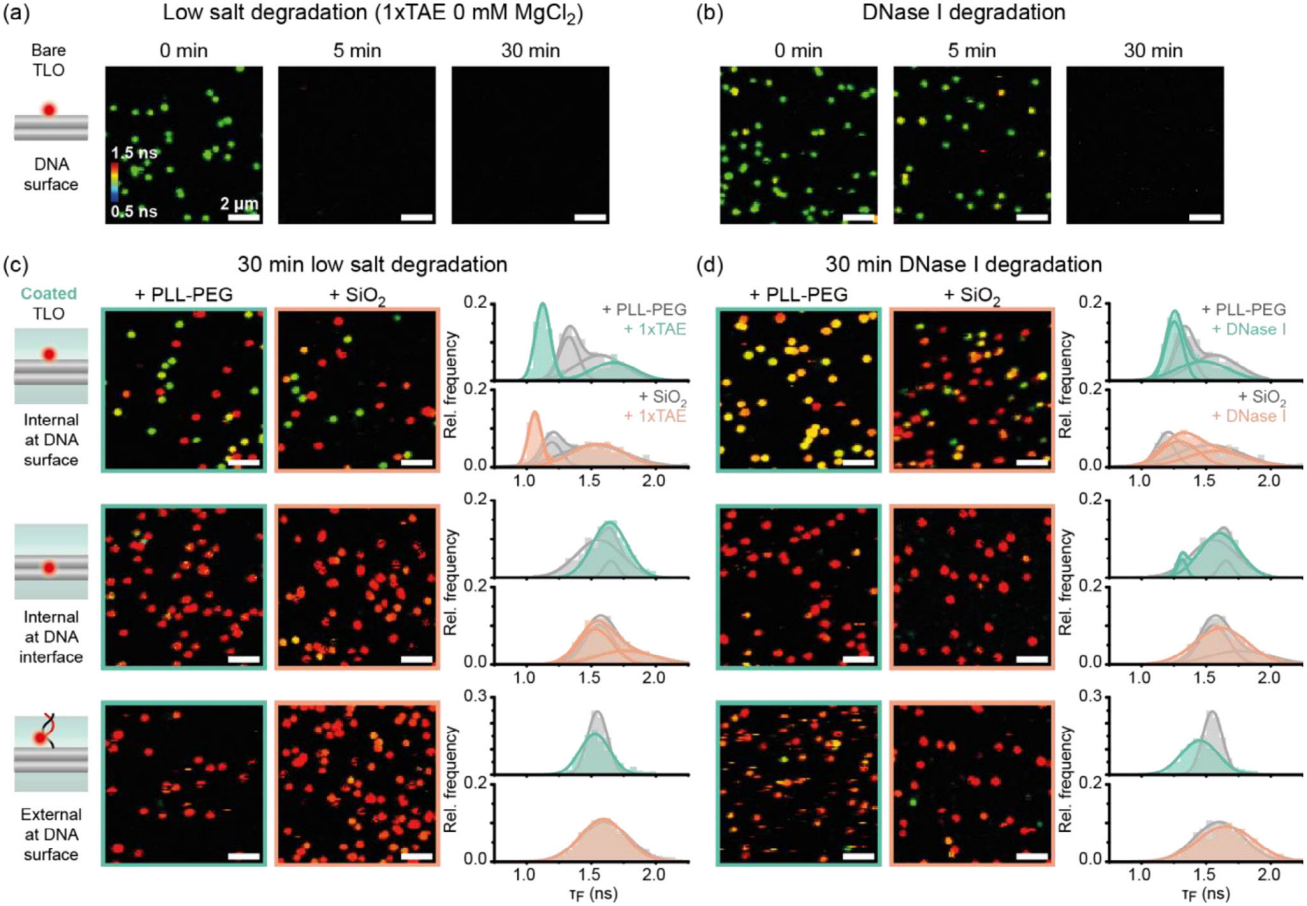
Using the fluorescence lifetime sensor to probe the stability of DNA origami structures and the coating layers in degrading conditions. a) Real‐time degradation study of uncoated TLO in Mg^2+^ free 1×TAE buffer. b) Real‐time degradation study of uncoated TLO in DNase I solution. c) FLIM scans and spot‐integrated fluorescence lifetime distributions for PLL‐PEG (green)‐ and silica (orange) coated TLOs after 30 min incubation in Mg^2+^ 1×TAE buffer. Sample sizes for PLL‐PEG coated TLOs top to bottom 756, 1059, and 908 molecules. For silica coated TLOs top to bottom 664, 1553, and 1847 molecules. d) FLIM scans and spot‐integrated fluorescence lifetime distributions for PLL‐PEG (green)‐ and silica (orange) coated TLOs after 30 min incubation in DNase I solution. Grey graphs represent fluorescence lifetime distributions of intact, coated TLO as reference. Sample sizes for PLL‐PEG coated TLOs top to bottom 1027, 1093, and 1160 molecules. For silica coated TLOs top to bottom 886, 622, and 816 molecules.

The quantitative reversibility of the molecular sensor enables not only the investigation of the integrity of the DNA nanostructure but also of the coating layers at a single nanostructure level and in real‐time. We thus next aimed to monitor the stability of the DNA origami and the coating layers in harsh and degrading conditions by first confirming that the observed fluorescence lifetime shifts correlate with the expected improved stability. To this end, we coated TLO labeled with AF647 at different positions with PLL‐PEG or silica and subsequent exposure the nanostructures to degrading conditions, i.e., Mg^2+^ free buffer containing EDTA (**Figure** **5**a, c) or a solution containing degrading enzyme (DNase I, Figure 5b,d). First, the qualitative degradation of uncoated TLO was probed in real‐time by applying the degrading solutions and subsequently acquiring FLIM images of the same field of view every 5 min (Figure S12, Supporting Information). Incubation of nanostructures in Mg^2+^ free buffer led to complete degradation of uncoated TLO within the first 5 min, while addition of DNase I degraded all nanostructures within the first 15 min, highlighting the low stability of uncoated DNA origami nanostructures in low‐salt conditions and in the presence of nucleases (Figure 5a,b and Figure S12a, Supporting Information). PLL‐PEG and silica coated structures, on the other hand, survived the incubation by either low salt buffers or DNase I over the full 30 min tested, indicating the successful stabilization by the protective coatings (Figure S12, Supporting Information). The apparent loss of FLIM labels over time was comparable to the observed photobleaching of an uncoated reference sample (Figure S13, Supporting Information). Since the coated nanostructures revealed increased fluorescence lifetimes even after 30 min incubation in degrading conditions, we concluded that a higher fluorescence lifetime of the sensor dye indeed goes hand in hand with successful coating and the effective stabilization of DNA origami nanostructures in degrading conditions.

To investigate the integrity of the coating layers further, we quantified the shift in fluorescence lifetime distributions of PLL‐PEG and silica coated TLOs after 30 min incubation in either Mg^2+^ free buffer containing EDTA or in the presence of DNase I (Figure 5c,d). In general, coated TLO nanostructures were not only stable over time in mild imaging buffer conditions, but also withstood the degrading conditions and preserved high fluorescence lifetime populations corresponding to an intact coating layer. Only the TLO with an internal AF647 label at the DNA surface exhibited a partial degradation of both the polymer and silica coating when incubated in a Mg^2+^‐free buffer (as indicated by the arising peak around 1.05 ns in fluorescence lifetime distribution previously assigned to uncoated TLO in Figure 3c, first row, see also Table S10, Supporting Information). As this labeling position is close to a folding defect we observe in a sub‐population of the TLO nanostructures (see Figure S14, Supporting Information), we reasoned that this lifetime population might indicate a point of weakness at this position. To test if the nanostructures contributing to this fluorescence lifetime population are still stabilized by a partially degraded coating or whether they are indeed uncoated and, thus, prone to degradation, we incubated the resulting sample additionally in DNase I solution. The second degradation step resulted in loss of nanostructures giving rise to fluorescence lifetimes around 1.05 ns, indicating that the observed lower fluorescence lifetime populations can be attributed to DNA origami structures that had compromised coating upon the first degradation (Figure S15, Supporting Information). These results highlight the sensitivity of the FLIM sensor and showcase its ability to probe the stability of different DNA origami coating agents in degrading conditions ultimately aiding the optimization of existing and the development of new protective strategies.

**Figure 6:**
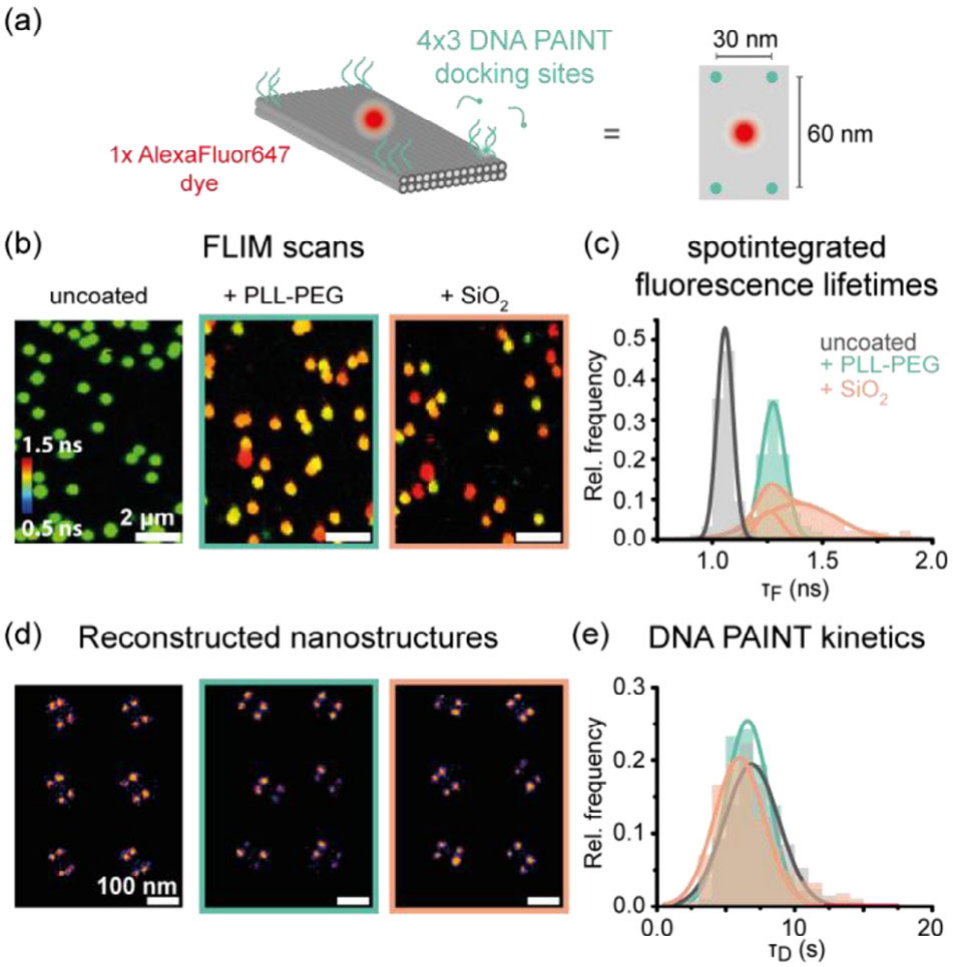
Combining the molecular sensor with DNA PAINT imaging for simultaneous investigation of the coating, structural integrity of the nanostructure, and addressability of DNA docking sites. a) Labeling scheme of a TLO internally labeled with AF647 at the upper surface and 4× three DNA PAINT docking sites in the corner regions of the TLO design. b) FLIM scans of uncoated, PLL‐PEG and silica coated TLO indicate successful coating. c) Spot‐integrated fluorescence lifetime distributions of uncoated, PLL‐PEG and silica coated TLO reveal a shift upon coating. d) Picked and aligned DNA PAINT images of uncoated, PLL‐PEG and silica coated TLO revealing the designed rectangular geometry. e) Extracted DNA PAINT dark time (τ_D_) distributions for uncoated, PLL‐PEG and silica coated TLO indicate similar accessibility for imager strands in solution. Sample sizes of 837 molecules for uncoated, 704 molecules for PLL‐PEG coated, and 571 molecules for silica coated TLOs.

Last, we combined our molecular sensor with DNA PAINT super‐resolution imaging to highlight its applicability for future correlative single‐molecule imaging techniques.^[^ ^44^ ^]^ To this end, we incorporated DNA PAINT docking sites in a rectangular pattern (lengths of ≈30 and 65 nm) into a TLO nanostructure internally labeled with an AF647 dye at the surface (**Figure** **6**a). This design allowed us to obtain complementary information about the coated nanostructures: while the FLIM sensor reports on the successful coating of the nanostructure, DNA PAINT imaging provides insights into the structural integrity of the DNA origami design and into the addressability of designed DNA docking sites. In this matter, we first acquired FLIM scans of uncoated and coated TLO nanostructures and subsequently performed DNA PAINT imaging of the same sample slides on a wide‐field microscope. While grid‐like microscope slides or correlative FLIM‐DNA PAINT microscopes enable the truly correlative imaging of individual nanostructures,^[^ ^45^, ^46^ ^]^ our experiment gave insight on the same sample slides without correlating the same field of views. A shift of the fluorescence lifetime distributions of PLL‐PEG and silica coated nanostructures indicated again the successful coating for both coating strategies (Figure 6b,c; fitted values in Table S11, Supporting Information). After acquiring FLIM scans, the imaging buffer was exchanged with a Cy3B labeled DNA PAINT imager solution and the same sample slides of uncoated or coated DNA origami were imaged at a different field of view on a total internal reflection fluorescence microscopy (TIRFM) widefield setup with 532 nm excitation to obtain super‐resolved DNA PAINT images (Figure 6d). Distance analysis of picked nanostructures revealed the same rectangular labeling pattern with comparable distances for both coating agents as for uncoated TLO, highlighting the structural integrity after the coating process (Figure S16, Supporting Information). Still, for both coating strategies a small subpopulation of a dual spot pattern was observed, which could be interpreted as folding of the TLO origami along its longer axis induced by the coating agent. From time to time, we observed this effect also in AFM imaging where a subpopulation of TLO nanostructures revealed a rod‐shaped geometry with an increased height (Figure S17, Supporting Information). Similar folding defects have been previously observed for the coating of monolayer DNA origami with PLL‐PEG but with higher efficiencies (>50%) due to the higher flexibility of the investigated nanostructure.^[^ ^28^ ^]^ By extracting the DNA PAINT dark times, i.e., the time between two binding events, the accessibility of the DNA PAINT docking sites on uncoated and coated nanostructures was compared. Both PLL‐PEG and silica coated TLO nanostructures revealed similar dark times as the uncoated TLO (Figure 6E, fitted values in Table S12, Supporting Information), highlighting the preserved addressability of DNA docking sites even after the formation of a protective layer.^[^ ^32^, ^33^ ^]^

## 3 Discussion

Our results demonstrate that the red cyanine dye AF647 is a suitable molecular FLIM sensor for probing the coating of DNA origami nanostructures in real time. However, the high sensitivity of the molecular sensor to its microenvironment makes it also susceptible to variations in fluorescence lifetime when labeled at different positions on the DNA origami. Consequently, for every labeling position, the sensor dye revealed a distinct fluorescence lifetime distribution before and after the coating which in turn can lead to varying fluorescence lifetime shifts. Nevertheless, we observed a pronounced absolute increase in the fluorescence lifetime in the range of 0.2 to 0.5 ns for all labeling positions, independent of the coating material.

To better understand the mechanistic origin of the observed fluorescence lifetime shift upon coating the DNA nanostructure, we carried out further mechanistic studies with the TLO origami labeled with an internal AF647 at the DNA surface. To quantify the sensitivity of the AF647 label on the DNA origami toward quenching by water, we measured the fluorescence lifetime in a D_2_O buffer (Figure S18, Supporting Information) and observed a shift of around 0.4 ns, similar to the observed fluorescence lifetime shifts upon coating with PLL‐PEG or silica. To further investigate the role of a restricted photoisomerization of the cyanine dye in the coating layer, we performed fluorescence intensity correlation analysis of single‐molecule trajectories of AF647 labeled DNA origami before and after coating (Figure S19, Supporting Information). While quenching by water only affects the S_1_ excited state of the fluorophore, a shift in the photoisomerization rate affects the photophysics of the dye (i.e., occurring dark states), which can be probed via intensity autocorrelation. To shed more light on two different fluorescence lifetime populations that were observed for coated DNA origami nanostructures, we grouped the intensity autocorrelation curves obtained from single‐molecule trajectories to two populations based on their fluorescence lifetimes as observed in Figure 2. Intensity autocorrelation curves obtained from PLL‐PEG coated TLOs with shorter fluorescence lifetime were comparable to those of uncoated TLOs indicating that the observed fluorescence lifetime shift is not related to the restricted photoisomerization or other effects on dye photophysics, but perhaps is predominantly induced by reduced water quenching. In the intensity autocorrelation curves of PLL‐PEG coated TLOs with higher fluorescence lifetime, we observed a slowed down photoisomerization with a reduced amplitude, indicating that the larger fluorescence lifetime shift could be additionally induced by restricted photoisomerization (for more detailed discussion, see Supplementary Note 1). Even though we found similar trends for silica coating, no significant difference between the lower and the higher fluorescence lifetime subpopulations was observed which we attributed to a dynamic interchange of the dye between two environments with different fluorescence lifetimes. This was also visible in continuous FLIM scans of the same field of view and far more pronounced for the silica coated nanostructures (Figure S20, Supporting Information). Altogether, the mechanistic studies suggest, that both effects, a restricted photoisomerization and reduced water quenching, contribute to the fluorescence lifetime shifts upon coating whereas the exact microenvironment around the dye within the coating layer determine which effect has a higher impact.

FLIM and DNA PAINT imaging on the same sample slides revealed a deformed subpopulation of the TLO nanostructures induced by PLL‐PEG and silica coating, as it has been reported previously for PLL‐PEG coated DNA monolayer origamis. This highlights that coating‐induced effects can lead to severe deformation even for rigid nanostructures and showcases that non‐invasive fluorescence‐based methods as the one reported here could be suitable tools to assess these deformations or artifacts induced by the coating agents, especially on a single nanostructure level.

While we performed FLIM measurements on an advanced single‐molecule microscope to study the coating layer on a single nanostructure level, one could also envision that ensemble lifetime readout could be used for a quick assessment of the coating of the nanostructures in solution. To further improve the fluorescence contrast of the molecular sensor, in the future one could explore a palette of alternative environment‐sensitive dyes, e.g. red‐emitting dyes such as Atto647N or Cy7 which have been reported to be quenched by water even more efficiently.^[^ ^39^ ^]^ Self‐blinking dyes like silica rhodamines, on the other hand, could potentially be applied to our sensor design to realize a fluorescence blinking or intensity‐based readout, enabling the probing of the coating layer also on widefield microscopes.^[^ ^47^ ^]^

## 4 Conclusion

In this work we exploited the environment‐sensitive cyanine dye AF647 to design a simple single‐molecule sensor to probe the protective coating of DNA origami nanostructures by either the block copolymer PLL‐PEG or by silica. By acquiring FLIM scans, the lifetime shift toward longer lifetimes can be utilized to screen the coating process qualitatively on a single nanostructure level, noninvasively and in real‐time. By placing the fluorophore at different positions on the DNA origami, the coating process and its effects on the nanostructure were investigated at the position of interest with nanometer precision. Further mechanistic studies suggested that both reduced quenching by water and restricted photoisomerization in the coating layer could induce the observed fluorescence lifetime increase of the employed sensor dye AF647. The reversibility of the molecular FLIM sensor could be exploited to follow the quantitative decomplexation of PLL‐PEG coating by dextran sulfate and to investigate the integrity of the coating in degrading conditions in real time. By combining the molecular sensor design with DNA‐PAINT we could probe for the first time the successful coating, structural integrity and addressability of DNA docking sites on the same sample slide, which was previously not possible due to the invasive nature of structural characterization methods, such as TEM or AFM. The sensor's broad applicability and consistent fluorescence lifetime shift across different coating materials make it a promising tool for investigating various coating strategies, such as encapsulation of DNA nanostructures with proteins^[^ ^48^ ^]^ or studying the coating of alternative substrate materials like lipid nanoparticles or metal‐organic frameworks. The noninvasive nature of the FLIM sensor approach, combined with its compatibility with other single‐molecule techniques like super‐resolution microscopy or fluorescence resonance energy transfer (FRET), enables the investigation of coatings in a wide range of applications, including drug delivery and release, biosensing, and biocomputing.

## 5 Experimental Section

### Materials

The p8064 scaffold strand used for the folding of the DNA origami nanostructures was extracted from M13mp18 bacteriophages (produced in‐house). Unmodified staple strands were purchased from Eurofins Genomics GmbH (Germany) and Integrated DNA Technologies (USA). Dye labeled oligonucleotides for DNA PAINT imaging or permanent labeling were purchased from Eurofins Genomics GmbH (Germany).

### DNA Origami Folding and Purification

All investigated TLO DNA origami nanostructures (also shown in Figure S1, Supporting Information) were folded in a 1× TE buffer containing 12 mM MgCl_2_ with a linear thermal annealing ramp from 60 to 44 °C with 1 h °C^−1^ after an initial 65 °C denaturation step. The 12HB DNA origami nanostructures were folded in a 1× TAE buffer containing 16 mM MgCl_2_ using the same scaffold strand as for the TLO. The structures were folded with a nonlinear thermal annealing ramp starting at 65 °C and then cooling down to 4 °C over a period of 25 h.^[^ ^49^ ^]^ Modifications of the DNA origami were realized using caDNAno (version 2.2.0). A full list of unmodified and modified staple strands and sequences are given in Tables S2–S4, S13 and S14 (Supporting Information). Folded DNA origami nanostructures were purified by filtration using Amicon Ultra filters (100 K, Merck, Germany). Concentrations of purified sample solution were measured via UV–vis spectroscopy (NanoDrop, Fischer Scientific, USA). More details on DNA origami design, folding, and purification procedures are given in Section 1.1 (Supporting Information).

### Sample Preparation

Cleaned high precision µm microscope cover glass (170 µm, 22 mm × 22 mm, No. 1.5H glass slides, Carl Roth GmbH, Germany) were assembled into inverted flow chambers as described previously.^[^ ^50^ ^]^ The assembled chambers were passivated with BSA‐biotin (Sigma Aldrich, USA) and functionalized with either neutravidin or streptavidin (Sigma Aldrich, USA). For immobilization, purified DNA origami was diluted to approximately 50 pM in 1× PBS buffer containing 500 mM NaCl and incubated in the chambers for ≈5 min and stored in a 1× TAE containing 10 mM MgCl_2_. Sufficient surface density was probed with a TIRF microscope. For more details on sample preparation, see Section 1.5 (Supporting Information).

### Coating with PLL‐PEG or Silica

The PLL‐PEG block copolymer K10PEG (1K) (Alamanda polymers, USA) was dissolved and stored in ultra‐pure water at a concentration of 2 mM.^[^ ^27^, ^28^ ^]^ Aliquots were stored at ‐20 °C and thawed and ultrasonicated for 10 min before use. To coat immobilized DNA origami nanostructures, the 2 mM PLL‐PEG solution was diluted in a 1× TAE buffer containing 10 mM MgCl_2_ to a final concentration of 20 µM and incubated for 30 min. To decomplex the cationic PLL‐PEG coating from the DNA origami, a 20 µM solution of anionic dextran sulfate (Sigma Aldrich, M = 20 000 g mol^−1^)^[^ ^28^ ^]^ in 1× TAE 10 mM MgCl_2_ was incubated in the coated sample chambers for 30 min. After washing, samples were then stored in 1× TAE containing 10 mM MgCl_2_. For the silicification of immobilized DNA origami an adapted version of the protocol of Fan and co‐workers was applied.^[^ ^26^, ^33^ ^]^ After hydrolysis of 100 µL TMAPS (50% (wt/wt) in methanol, TCI America) in 5 mL 1× TAE (40 mM Tris, 2 mM EDTA, 12.5 mM MgAc_2_, pH = 8.0) for 20 min under vigorous stirring, 100 µL TEOS (98%, Sigma Aldrich, USA) was added and stirred for another 20 min. Freshly prepared precursor solution was incubated for 24 h. The coated sample chambers were washed with 80% ethanol and with ultra‐pure water. The samples were then stored in 1× TAE containing 10 mM MgCl_2_. For AFM imaging, mica slides with immobilized DNA origami were analogously incubated with either 20 µM PLL‐PEG solution or with freshly prepared silica precursor solution. For more details on coating of immobilized DNA nanostructures, see Section 1.6 (Supporting Information).

### Degradation Studies

To probe the stability of coated and bare DNA origami in degrading conditions, either a low‐salt buffer or a DNase I solution were incubated on immobilized nanostructures. Magnesium ion free conditions were realized by incubation of a 1× TAE buffer on surface immobilized nanostructures for 30 min. Enzymatic degradation of immobilized DNA origami nanostructures was tested by incubation of a DNase I solution (1:10 dilution of DNase I (1 U µl^−1^) in 1× TAE containing 10 mM MgCl_2_, Thermo Fisher Scientific, USA) for 30 min.

### AFM Imaging

AFM scans in aqueous solution (AFM buffer = 40 mM Tris, 2 mM EDTA, 12.5 mM Mg(OAc)_2_·4 H_2_O) were performed on a NanoWizard^®^ 3 ultra AFM (JPK Instruments AG). Measurements were performed in AC mode on a scan area of 3×3 µm with a micro cantilever (νres = 110 kHZ, kspring = 9 N m^−1^, Olympus Corp.). Leveling, background correction and extraction of height histograms of obtained AFM images were realized with the software Gwyddion (version 2.60).^[^ ^51^ ^]^ For more details on sample preparation and AFM imaging, see Section 1.4 (Supporting Information).

### Confocal Microscopy

Fluorescence lifetime imaging microscopy (FLIM) and intensity autocorrelation studies were performed on a home‐built confocal microscope based on an Olympus IX‐71 inverted microscope as described previously.^[^ ^52^ ^]^ AF647 modifications labeled to surface‐immobilized DNA origami were excited by a pulsed 640 nm excitation at a repetition rate of 40 MHz. The setup was controlled by a commercial software package (SymPhoTime64, PicoQuant GmbH, Germany). For more details on FLIM imaging and intensity autocorrelation studies, see Section 1.8 (Supporting Information).

### Wide‐Field Microscopy

DNA‐PAINT measurements were carried out on a commercial Nanoimager S (ONI Ltd., UK). Red excitation at 640 nm was realized with a 1100 mW laser, green excitation at 532 nm with a 1000 mW laser, respectively. For imaging, a 1× PBS buffer containing 500 mM NaCl and an imager concentration of 5 nM was used. The 8 nt imager oligonucleotide with a Cy3B label on the 3′‐end was purchased from Eurofins Genomics GmbH (Germany) and consisted of the sequence 5′‐GGAATGTT‐3′. Acquired DNA‐PAINT raw data were analyzed using the Picasso software package.^[^ ^50^ ^]^ After drift correction, individual DNA nanostructures were picked, aligned and corresponding blinking kinetics extracted for further analysis. Distance analysis of obtained DNA PAINT images was performed with a custom written Python code. For more details on DNA PAINT imaging and data analysis, see Section 1.11 (Supporting Information).

### Statistical Analysis

Presented FLIM images were exported from the acquisition software with a fixed brightness contrast for illustrative purpose (0 to 100 photons/px). Acquired FLIM scans (.ptu files) were analyzed with a custom‐written Python software. For more details on FLIM analysis, see Section 1.10 (Supporting Information) for spot‐integrated fluorescence lifetimes and Section 1.11 (Supporting Information) for re‐convoluted fluorescence lifetimes. Spot‐integrated or re‐convoluted fluorescence lifetime values were plotted as histograms with bin sizes of 0.05 ns and fitted with Gaussian distributions using OriginPro 2019 (OriginLab, USA).

## Conflict of Interest

The authors declare no conflict of interest.

## Author Contributions

M.S. and G.A.B. contributed equally to this work. V.G., M.S., and A.H.‐J. conceived the idea. G.A.B. designed the TLO origami. M.S. and G.A.B. fabricated all samples and carried out AFM and FLIM imaging experiments, single‐molecule intensity autocorrelation studies, and data analysis. T.S. contributed to the mechanistic intensity autocorrelation studies. K.B. and L.M.W. contributed to the silica coating experiments. M.S. and G.A.B. prepared figures. V.G., A.H.‐J. and P.T. supervised the study. M.S., V.G. and G.A.B. wrote the manuscript with contributions from all authors. All authors have given approval to the final version of the manuscript.

## Supporting information



Supporting Information

## Data Availability

The data that support the findings of this study are available from the corresponding author upon reasonable request.
